# Comprehensive analysis of CXCL14 uncovers its role during liver metastasis in colon cancer

**DOI:** 10.1186/s12876-023-02896-z

**Published:** 2023-08-10

**Authors:** Lei Zhou, Yan Zhang, Ming Wei, Kangming Du, Jing Lin, Lihong Wei

**Affiliations:** 1 Pain Management, Hospital of Chengdu University of Traditional Chinese Medicine, Chengdu, Sichuan China; 2 Gastroenterology Department, Hospital of Chengdu University of Traditional Chinese Medicine, Chengdu, Sichuan China; 3 Department of Gynecology, Hospital of Chengdu University of Traditional Chinese Medicine, Chengdu, Sichuan China; 4 Department of Vascular Surgery, Hospital of Chengdu University of Traditional Chinese Medicine, Chengdu, Sichuan China; 5 Department of Anesthesiology operating room, Hospital of Chengdu University of Traditional Chinese Medicine, Chengdu, Sichuan China; 6 Department of Tuina, Hospital of Chengdu University of Traditional Chinese Medicine, Chengdu, Sichuan China

**Keywords:** colon cancer, CXCL14, Liver metastasis, Fibroblasts, Prognosis

## Abstract

**Background:**

The most common cause of death for colon cancer patients is liver metastasis.

**Methods:**

All the data enrolled in this study were downloaded from two public databases, The Cancer Genome Atlas Program, the TCGA-COAD project and Gene Expression Omnibus, GSE41258 project. All the analysis was performed in R software.

**Results:**

In our study, we systematically explored the molecules involved in the liver metastasis process of colon cancer. The biological role of these molecules was identified through the GO and KEGG analysis. Moreover, we identified that the molecules SERPINA3, SERPINA1, MMP3, ALDH1A3, PBK and CXCL14 were the independent factors for patients survival. The CXCL14 was selected for further analysis for its most significant P value. Single-cell analysis showed that the CXCL14 was mainly expressed in the fibroblasts. Meanwhile, the biological role of fibroblasts in the colon cancer microenvironment was investigated. Further, the clinical role of CXCL14 in colon cancer was also explored. The result showed that the CXCL14 is a protective factor against colon cancer independent of other clinical parameters like age, gender, clinical stage, and TNM classifications. Then, biological enrichment analysis indicated that the CXCL14 is predominantly involved in the activating of the WNT/β/catenin pathway, pancreas beta cells, peroxisome and bile acid metabolism. Immune infiltration analysis showed that for the patients with high CXCL14 levels, the plasma B cells, CD8 + T cells, neutrophil and NK cells might infiltrate more, in contrast to B cells, monocyte and macrophages. Furthermore, we found that the patients with low CXCL14 expression might be more sensitive to etoposide, rapamycin and sunitinib.

**Conclusions:**

Our result could improve the understanding of the liver metastasis process in colon cancer. Also, CXCL14 was identified as an underlying therapeutic target for colon cancer.

**Supplementary Information:**

The online version contains supplementary material available at 10.1186/s12876-023-02896-z.

## Introduction

Globally, colon cancer is one of the most common digestive cancers, and its incidence is projected to increase [1]. Over 1.9 million incidences and 0.9 million deaths occurred worldwide in 2020 [2]. It is estimated that 2.2 million new cases of colorectal cancer will occur by 2030, as well as 1.1 million deaths from colorectal cancer [3]. The heterogeneity of colon cancer contributes to variable diagnosis, which results in a low 5-year survival rate for patients with advance-stage metastatic disease and a high recurrence rate [4, 5]. In addition, colon cancer is characterized by rapid progression and metastasis that results in a variable prognosis for patients [6]. Analysis of gene expression variation and molecular risk stratifications of colon cancer can provide important insight into the molecular mechanisms that lead to its initiation and development [7]. It is necessary to develop an accurate model of colon cancer risk and to determine molecular biomarkers for the disease.

The 5-year survival rate of colon cancer patients has improved with advances in surgical and medical therapies, but nearly half of the colon cancer patients suffer from distant metastasis, which is the primary cause of treatment failure [8]. It has been reported that only 8.1% of colon cancer patients with metastasis will survive five years after diagnosis [9]. Approximately 90% of those who die from colon cancer do so as a result of its metastatic abilities and distant invasive capabilities [10]. Primary and metastatic sites of colon cancer have different epidemiological, biological, and clinical characteristics, suggesting that different molecular mechanisms underlie carcinogenesis and progression [11]. Because liver metastasis from colon cancer is the most frequent form of distant metastasis, understanding the mechanism is crucial for improving disease control in the future [12]. Liver metastasis in colon cancer can be partially explained by the mesenteric veins joining the splenic veins to form a portal system that flows directly into the liver. The crucial molecular step in the development of colon cancer distant metastasis contains epithelial-mesenchymal transition (EMT), and the loss of cellular adhesion proteins such as E-cadherin and tight junction proteins [13]. Higher levels of E-cadherin expression have been reported to correlate with longer overall survival (OS) in colon cancer liver metastasis [14]. Thus, further studies must be conducted on molecular mechanisms for colon cancer liver metastasis as well as more biomarkers [15].

Large volumes of genomic data have been generated by next-generation sequencing technologies, which provide convenience for researchers []. Here, we systematically explored the molecules involved in the liver metastasis process of colon cancer. The CXCL14 was finally identified. Then, the single-cell analysis, biological enrichment, immune infiltration and drug sensitivity analysis were performed to illustrate the role of CXCL14 in colon cancer, which enriched its regulatory effect in cancers.

## Methods

### Public data collection

The public data used for this study was collected from The Cancer Genome Atlas Program (TCGA) database, the TCGA-COAD project and Gene Expression Omnibus (GEO) database, GSE41258 project. For the TCGA database, the transcriptional profiling data was downloaded from the portal named TCGA-GDC in a “STAR-Counts” form. The R code was utilized for data induction. The Human reference file GRCh38.p13.gtf file was used for genomic probe annotation. Missing value completion and duplicate value merge were then performed to improve the data quality. The genes with median values less than 0.1 were removed. For clinical data, the initiative file form was “bcr-xml” and collated using the Perl code. For the data from the GSE41258 project, the original data was downloaded from the “Series Matrix File(s)” link and collated using the Perl code. The baseline information of TCGA-COAD patients was shown in Table 1.

**Table 1:** The baseline information of included patients

Clinical features		Number	Percentage (%)
Age	<=65	185	40.9
	> 65	267	59.1
Gender	Female	214	47.3
	Male	238	52.7
Stage	Stage I	76	16.8
	Stage II	178	39.4
	Stage III	125	27.7
	Stage IV	62	13.7
	Unknown	11	2.4
Tstage	T1	10	2.2
	T2	77	17.0
	T3	308	68.1
	T4	56	12.4
	Tis	1	0.2
Mstage	M0	334	73.9
	M1	62	13.7
	Unknown	56	12.4
Nstage	N0	269	59.5
	N1	103	22.8
	N2	80	17.7

### Biological enrichment

Gene Ontology (GO) and Kyoto Encyclopedia of Genes and Genomes (KEGG) analysis was conducted using the clusterprofiler package [16]. Furthermore, the gene set enrichment analysis (GSEA) analysis was utilized to identify the biological differences between two specific groups [17]. The reference gene sets were Hallmark and GO.

### Protein-protein interaction (PPI) network

The data for the construction of the PPI network was collected from the online STRING database [18]. The detailed parameters were as follows: the “meaning of network edges” was “evidence”; the “network type” was “full STRING network”. The Cytoscape software was utilized for network visualization.

### Prognosis analysis

The genes were combined with patients prognosis information and then the univariate Cox regression analysis was utilized to identify the molecules significantly associated with overall survival (OS, P < 0.05). Then, the identified genes were optimized using the LASSO regression algorithm. Next, multivariate Cox regression analysis was used to screen the independent prognosis markers.

### Analysis of genes at the single-cell level

The single-cell analysis was performed using the open data from the Tumor Immune Single-cell Hub (TISCH) project (http://tisch.comp-genomics.org/home/) [19].

### Immune relation analysis

Multiple algorithms, CIBERSORT, EPIC, MCPCOUNTER, QUANTISEQ, TIMER and XCELL were utilized to quantify the immune cell infiltration in COAD immune microenvironment [20–23]. The immune function of the individual sample was quantified using the single sample GSEA (ssGSEA) algorithm [24].

### Immunohistochemistry (IHC)

The representative IHC image of CXCL14 in colon cancer normal and tumor tissue was obtained from the Human Protein Atlas (HPA) database [25].

### Drug sensitivity analysis

The drug sensitivity analysis of CXCL14 was performed based on the data from the Genomics of Drug Sensitivity in Cancer (GDSC) database [26].

### Statistical analysis

All the statistical analysis were performed using the R version 4.0.0 software. Detailed, the threshold set for statistical significance was 0.05, which was two-sided. Different analysis methods are adopted according to different data distribution forms. Differentially expressed genes (DEGs) analysis was performed using the limma package. Kaplan-Meier (KM) survival curves were used to compare the prognosis difference between the two groups.

## Results

### Identification of the molecules involved in the liver metastasis process of colon cancer

**Fig. 1:**
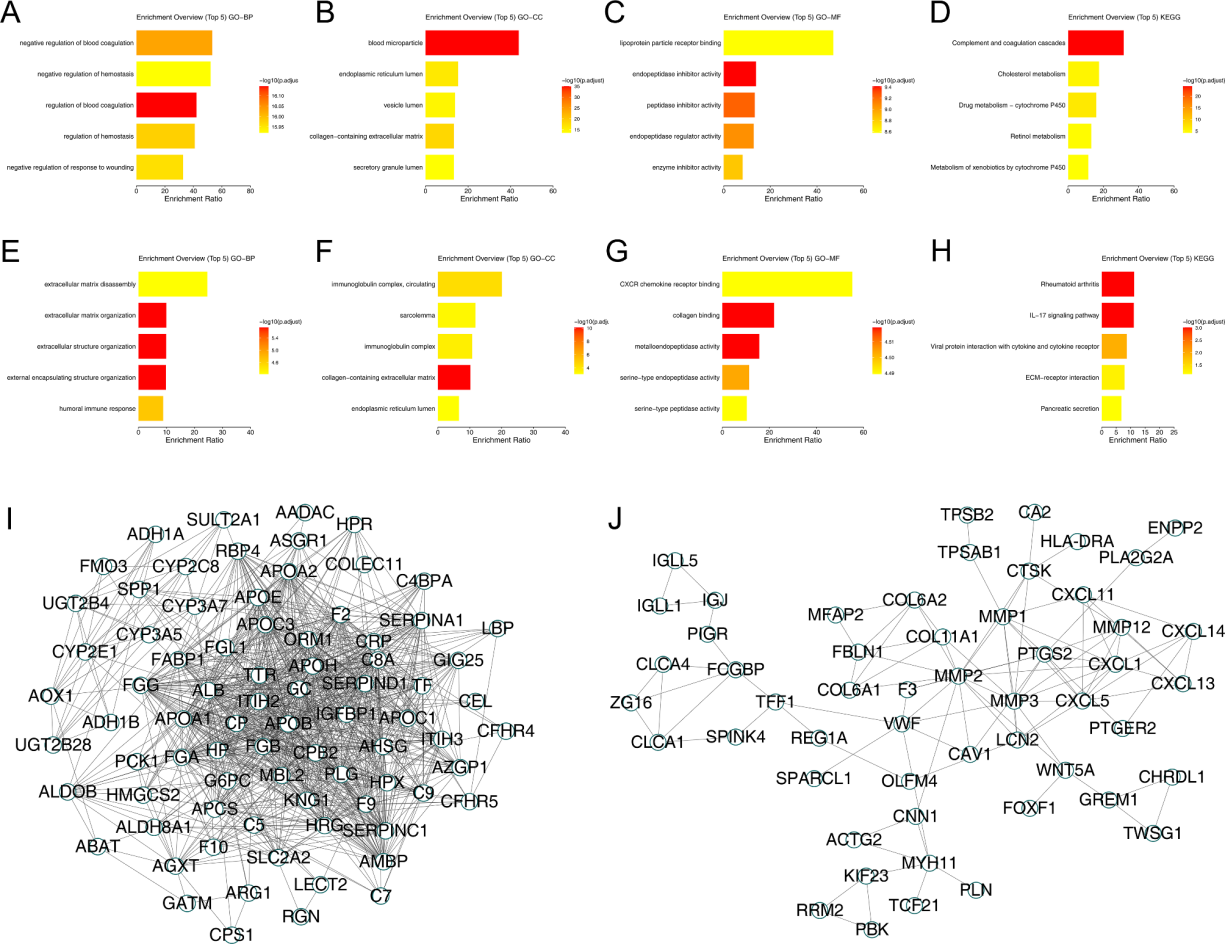
Biological enrichment analysis identified DEGs between primary and liver metastasis colon cancer tissue Notes: **A**: GO-BP analysis of upregulated DEGs; **B**: GO-CC analysis of upregulated DEGs; **C**: GO-MF analysis of upregulated DEGs; **D**: KEGG analysis of upregulated DEGs; **E**: GO-BP analysis of downregulated DEGs; **F**: GO-CC analysis of downregulated DEGs; **G**: GO-MF analysis of downregulated DEGs; **H**: KEGG analysis of downregulated DEGs; **I**: PPI network of upregulated genes; **J**: PPI network of downregulated genes

The flowchart of whole study was shown in Figure S1. The GSE41258 provided the transcription profile data of primary focus and liver metastasis tissue of colon cancer. The data preprocessing process was shown in Figure S2. Then, the limma package was utilized to conduct the DEG analysis between primary focus and liver metastasis colon cancer tissue. Totally, 84 genes were upregulated and 83 genes were downregulated were identified in the liver metastasis colon cancer tissue compared with the control tissue (Figure S3). For the upregulated genes, biological enrichment analysis showed that these molecules were mainly involved in the negative regulation of blood coagulation, negative regulation of hemostasis, regulation of blood coagulation, regulation of hemostasis and negative regulation of response to wounding (Fig. 1A, GO-BP); for GO-CC, the top five enriched terms were blood microparticle, endoplasmic reticulum lumen, vesicle lumen, collagen-containing extracellular matrix, secretory granule lumen (Fig. 1B, GO-CC); for GO-MF, the top five enriched terms were lipoprotein particle receptor binding, endopeptidase inhibitor activity, peptidase inhibitor activity, endopeptidase regulator activity and enzyme inhibitor activity (Fig. 1C, GO-MF); for KEGG, the top five enriched terms were complement and coagulation cascades, cholesterol metabolism, drug metabolism-cytochrome P450, retinol metabolism and metabolism of xenobiotics by cytochrome P450 (Fig. 1D, KEGG). The downregulated genes were mainly enriched in the extracellular matrix disassembly, extracellular matrix organization, extracellular structure organization, external encapsulating structure organization and humoral immune response (Fig. 1E, GO-BP); for GO-CC, the top five enriched terms were immunoglobulin complex-circulating, sarcolemma, immunoglobulin complex, collagen-containing extracellular matrix and endoplasmic reticulum lumen (Fig. 1F, GO-CC); for GO-MF, the top five enriched terms were CXCR chemokine receptor binding, collagen binding, metalloendopeptidase activity, serine-type endopeptidase activity and serine-type peptidase activity (Fig. 1G, GO-MF); for KEGG, the top five enriched terms were rheumatoid arthritis, IL-17 signaling pathway, viral protein interaction with cytokine and cytokine receptor, ECM-receptor interaction and pancreatic secretion (Fig. 1H, KEGG). Moreover, the PPI network was constructed to illustrate the underlying protein interaction. The network of upregulated genes was illustrated in Fig. 1I and the network of downregulated genes was shown in Fig. 1J.

### Screening of the molecules significantly affects patients survival

**Fig. 2:**
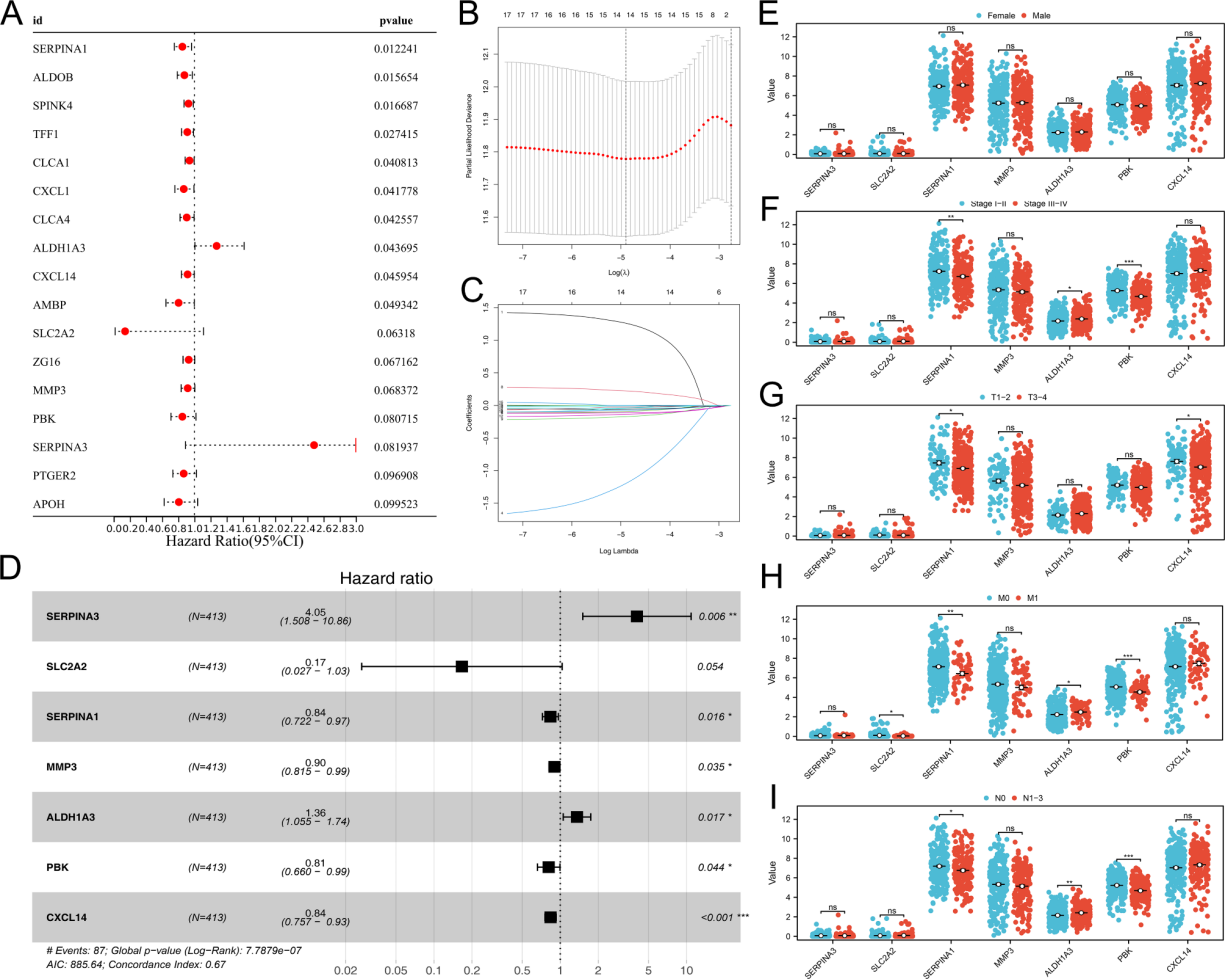
Prognosis model based on DEGs. Notes: **A**: Univariate Cox regression analysis based on identified DEGs; **B-C**: LASSO regression analysis; **D**: Multivariate Cox regression analysis; **E**: The expression level of SERPINA3, SLC2A2, SERPINA1, MMP3, ALDH1A3, PBK, CXCL14 in female and male patients; **F**: The expression level of SERPINA3, SLC2A2, SERPINA1, MMP3, ALDH1A3, PBK, CXCL14 in Stage III-IV and Stage I-II patients; **G**: The expression level of SERPINA3, SLC2A2, SERPINA1, MMP3, ALDH1A3, PBK, CXCL14 in T1-2 and T3-4 patients; **H**: The expression level of SERPINA3, SLC2A2, SERPINA1, MMP3, ALDH1A3, PBK, CXCL14 in M0 and M1 patients; **I**: The expression level of SERPINA3, SLC2A2, SERPINA1, MMP3, ALDH1A3, PBK, CXCL14 in N1-3 and N0 patients

Based on the genes identified above, we tried to identify the molecules involved in the process of colon cancer liver metastasis, as well as remarkably affecting patients prognosis. Univariate Cox regression analysis was conducted and the genes with P < 0.1 were regarded as the molecules significantly affecting patients survival (Fig. 2A). LASSO regression analysis was used to optimize data dimension (Fig. 2B-C). Multivariate Cox regression analysis indicated that the molecules SERPINA3, SERPINA1, MMP3, ALDH1A3, PBK and CXCL14 were the independent factors for patients survival (Fig. 2D). Clinical correlation showed no significant difference in these genes between female and male patients (Fig. 2E); SERPINA1 and PBK were downregulated, while ALDH1A3 was upregulated in stage III-IV patients compared with stage I-II patients (Fig. 2F); SERPINA1 and CXCL14 were downregulated in the T3-4 patients compared with the T1-2 patients (Fig. 2G); SERPINA1 was downregulated, while ALDH3A1 was upregulated in the M1 patients compared to the M0 patients (Fig. 2H); SERPINA1 and PBK were downregulated, while ALDH1A3 was upregulated in N1-3 patients compared with N0 patients (Fig. 2I).

### Single-cell analysis of CXCL14

**Fig. 3:**
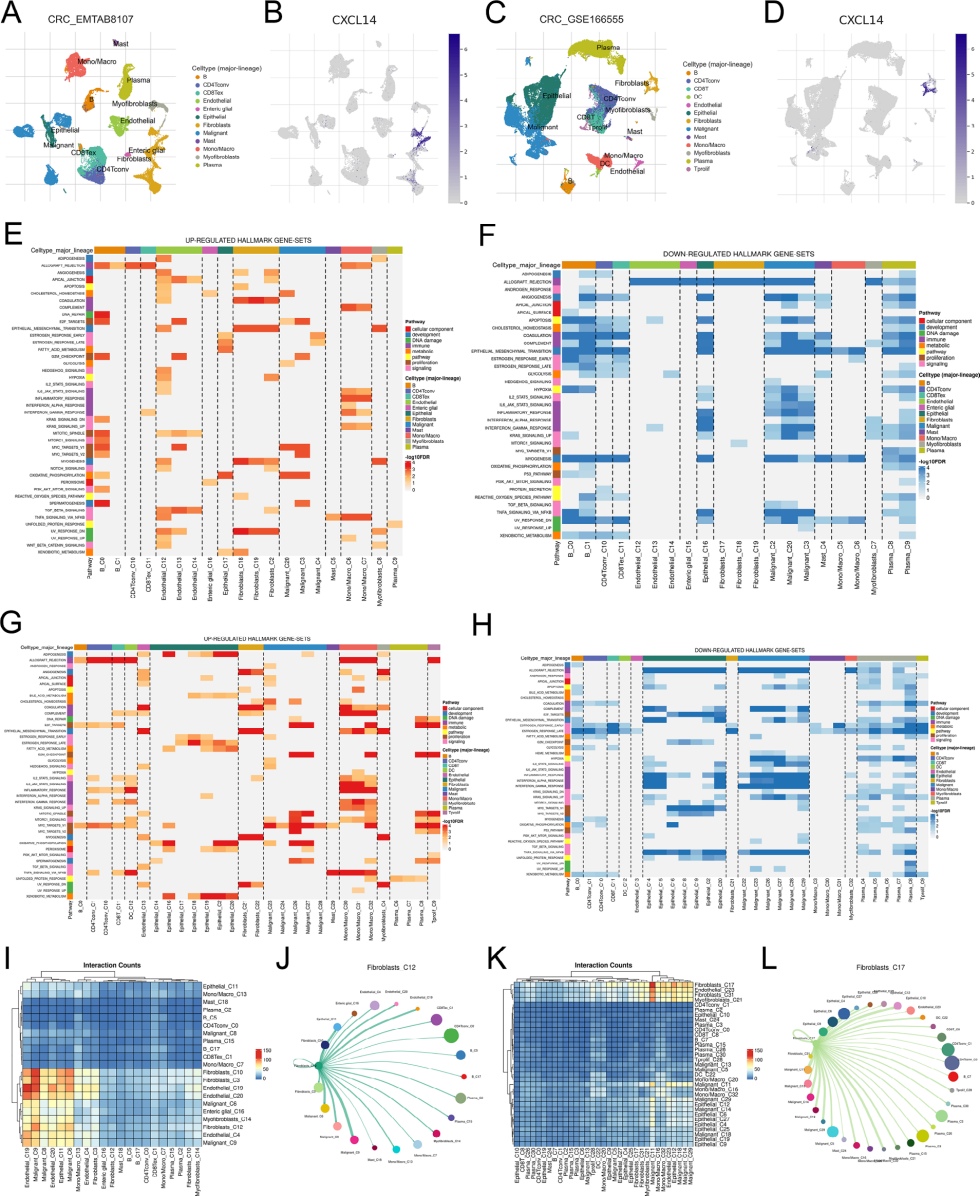
Single-cell analysis of CXCL14 and role of fibroblasts in colon cancer microenvironment Notes: **A-B**: The expression pattern of CXCL14 in single-cell cohort EMTAB8107; **C-D**: The expression pattern of CXCL14 in single-cell cohort GSE166555; **E**: The upregulated Hallmark terms fibroblasts involved in (EMTAB8107 cohort); **F**: The downregulated Hallmark terms fibroblasts involved in (EMTAB8107 cohort); **G**: The upregulated Hallmark terms fibroblasts involved in (GSE166555 cohort); **H**: The downregulated Hallmark terms fibroblasts involved in (GSE166555 cohort); **I-J**: The cell interaction between fibroblasts and other cells in EMTAB8107 cohorts; **K-L**: The cell interaction between fibroblasts and other cells in GSE166555 cohorts

CXCL14 has the most significant P value in multivariate Cox regression analysis and therefore was selected for further analysis. Based on the open-accessed data of CRC_EMTAB8107 and CRC_GSE166655, we investigated the gene expression pattern of CXCL14 in the colon cancer microenvironment. The results showed that in both CRC_EMTAB8107 and CRC_GSE166655 cohorts, CXCL14 was mainly expressed in the fibroblasts (Fig. 3A-D). Next, we explored the role of fibroblasts in the colon cancer microenvironment. GSEA analysis based on the Hallmark gene set indicated that fibroblasts are associated with a higher activity of coagulation, epithelial-mesenchymal transition (EMT), myogenesis and UV response DN, yet a lower activity of allograft rejection (Fig. 3E-F). Meanwhile, GSEA analysis based on the KEGG gene set showed that the fibroblasts are associated with a higher activity of angiogenesis, coagulation, complement, EMT, myogenesis, UV response DN, while a lower activity of estrogen response early and estrogen response late (Fig. 3G-H). In the CRC_EMTAB8107 cohort, the fibroblasts interacted closely with the endothelial and malignant cells (Fig. 3I-J). In the CRC_GSE166655 cohort, the fibroblasts interacted closely with the endothelial, epithelial and malignant cells (Fig. 3K-L).

### The clinical pattern of CXCL14

**Fig. 4:**
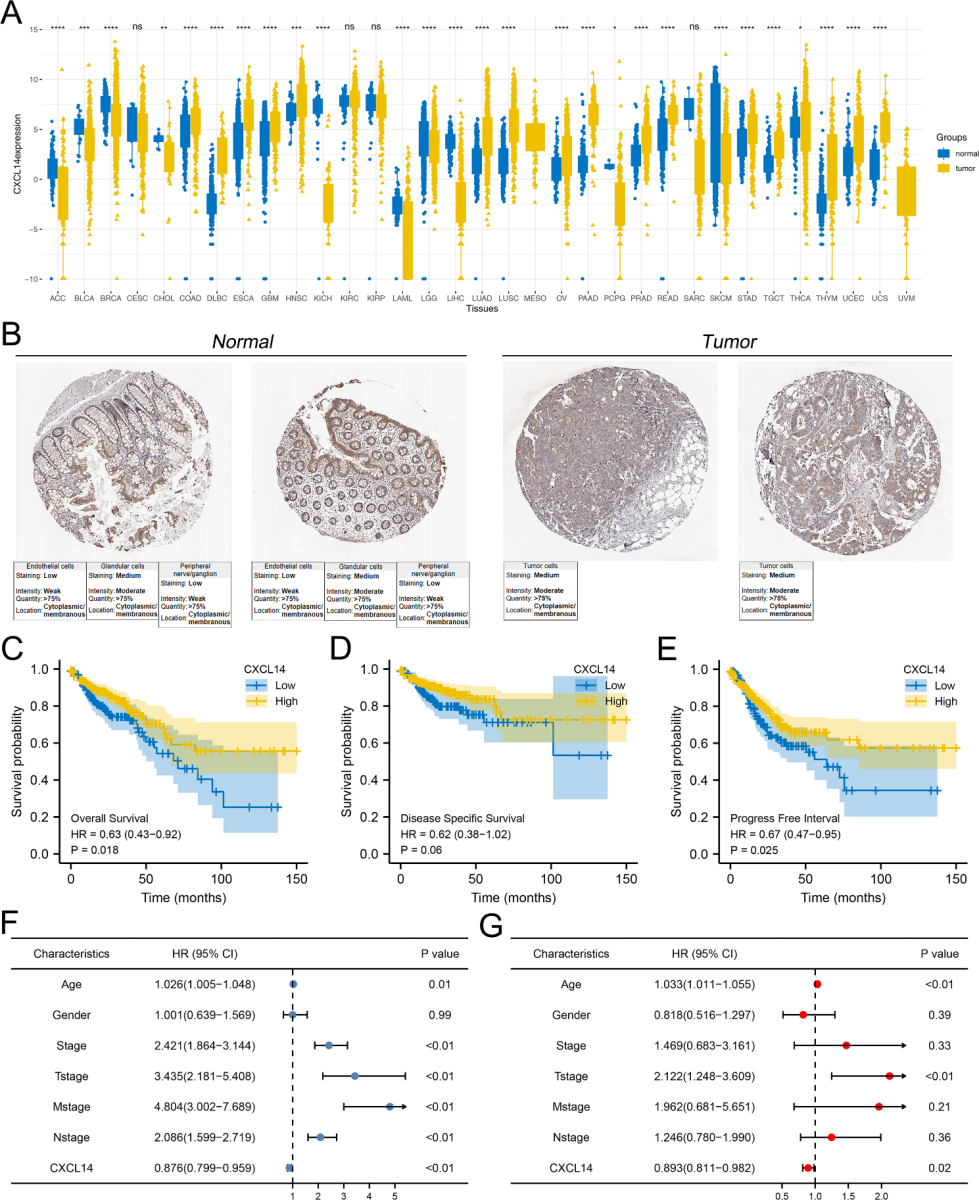
Expression pattern and clinical correlation of CXCL14 in colon cancer Notes: **A**: The pan-cancer expression pattern of CXCL14; **B**: The immunohistochemistry illustrating the protein level of CXCL14 between colon cancer and normal tissue; **C**: KM survival curves of OS in patients with high and low CXCL14 expression; **D**: KM survival curves of DSS in patients with high and low CXCL14 expression; **E**: KM survival curves of PFS in patients with high and low CXCL14 expression; **F**: Univariate Cox regression analysis of CXCL14 and other clinical features; **G**: Multivariate Cox regression analysis of CXCL14 and other clinical features

Pan-cancer analysis indicated that the CXCL14 was differentially expressed in most cancers and corresponding normal tissue, indicating its widespread effect on cancer development (Fig. 4A). Based on the data from TCGA-COAD and GTEx database, we noticed a higher RNA level of CXCL14 in colon cancer compared to the normal tissue (Fig. 4A). Nevertheless, the protein level of CXCL14 between colon cancer and normal tissue seemed to have no significant difference based on the data from the HPA database, indicating the underlying epigenetic regulation (Fig. 4B). KM survival curves indicated that the patients with higher CXCL14 expression levels might have worse overall survival (OS), disease-free survival (DSS) and progression-free survival (PFI) (Fig. 4C-E, OS, HR = 0.63, P = 0.018; DSS, HR = 0.62, P = 0.06; PFI, HR = 0.67, P = 0.025). Univariate and multivariate analysis revealed that the CXCL14 is a prognosis-protective marker independent of multiple clinical parameters like age, gender, and clinical and TNM classification (Fig. 4F-G).

### Pathway enrichment analysis

**Fig. 5:**
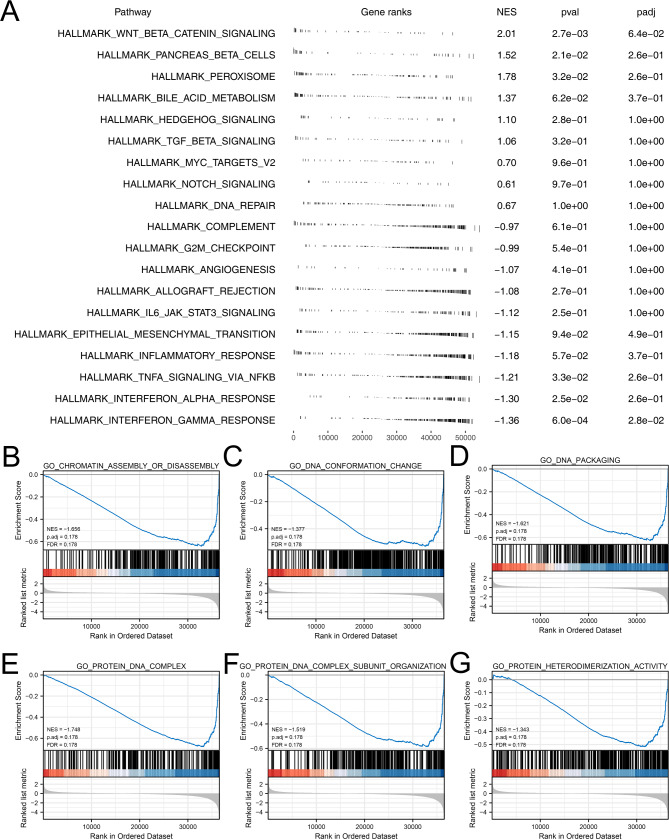
Biological enrichment analysis of CXCL14 Notes: **A**: GSEA analysis based on Hallmark gene set of CXCL14; **B-G**: GSEA analysis based on GO gene set

Following, we tried to explore the biological effect of CXCL14 exerting in colon cancer. GSEA analysis based on the Hallmark gene set showed that CXCL14 is predominantly involved in the activating of the WNT/β-catenin pathway, pancreas beta cells, peroxisome and bile acid metabolism (Fig. 5A). The GSEA analysis based GO gene set indicated that the CXCL14 can significantly weaken the activity of chromatin assembly or disassembly, DNA conformation change, NDA packaging, protein DNA complex, protein DNA complex subunit organization and protein heterodimerization activity (Fig. 5B-G).

### Immune infiltration and drug sensitivity analysis

**Fig. 6:**
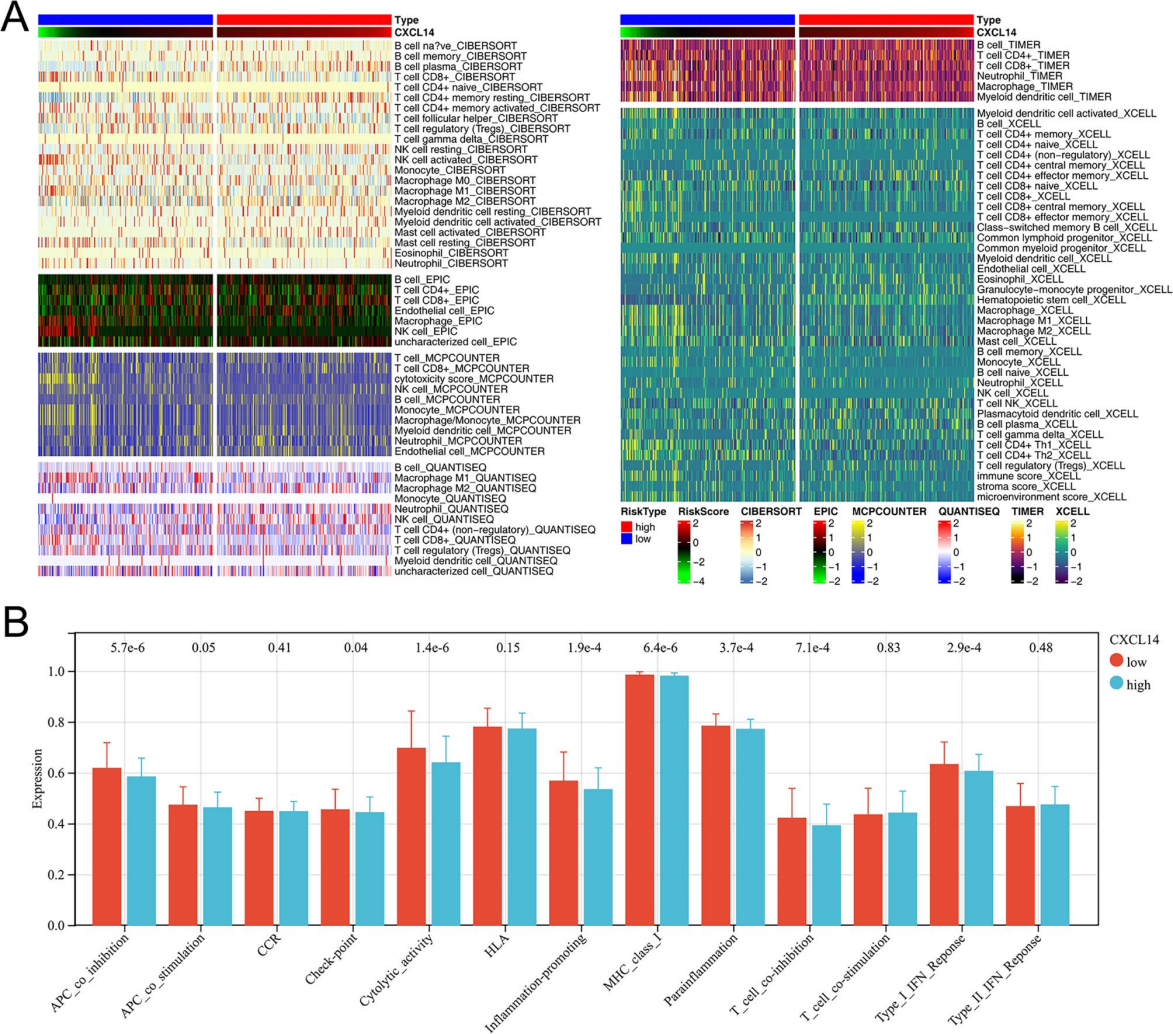
Immune cell and immune function analysis of CXCL14 Notes: **A**: The correlation between CXCL14 and the immune cell quantified by multiple algorithms; **B**: The immune function quantified by ssGSEA algorithm between high and low CXCL14 patients

**Fig. 7:**
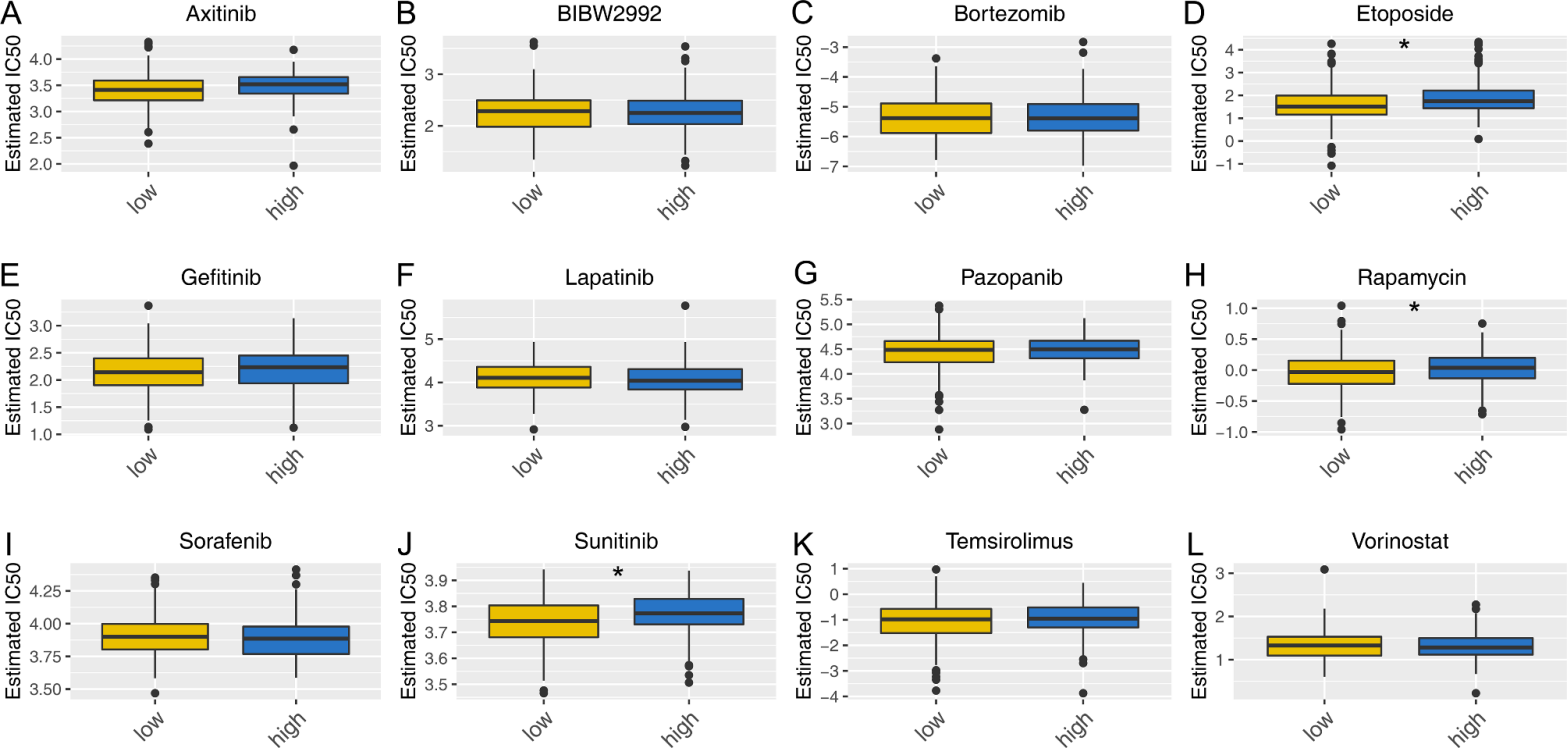
Drug sensitivity analysis Notes: **A-L**: The IC50 of specific drugs in patients with high and low CXCL14 level

Different cells exert a diverse role in the tumor microenvironment and influence cancer progression in multiple manners. We next explored the correlation between CXCL14 and different cells in the tumor microenvironment. Results showed for the patients with high CXCL14 levels, the plasma B cells, CD8 + T cells, neutrophil and NK cells might infiltrate more, in contrast to B cells, monocyte and macrophages (Fig. 6A). Additionally, we observed lower activity of APC_co_inhibiton, APC_co_stimulation, check-point, cytolytic activity, inflammation-promoting, T cell co_inhibition, type I IFN response in patients with high CXCL14 expression (Fig. 6B). Drug sensitivity analysis indicated that the patients with low CXCL14 expression might be more sensitive to etoposide, rapamycin and sunitinib (Fig. 7A-L).

## Discussion

Besides being the third most common cancer in the world, colon cancer is the second leading cause of cancer-related death [27]. Although colon cancer patients who are diagnosed at an early stage have better survival rates thanks to more effective and less toxic treatments, 30–50% of patients develop distant metastasis or recurrence within five years of treatment [28]. Advances in pathophysiological evaluation, treatment decisions, and prognostic predictions for colon cancer based on TNM staging systems and molecular markers require more investigation into effective diagnostic and prognostic biomarkers [29, 30]. Furthermore, recent immunotherapies targeting specific immune checkpoints such as PD-1/L1 and CTLA4 have shown promising results in colon cancer treatment, suggesting that the tumor microenvironment plays an important role [31, 32].

In our study, we systematically explored the molecules involved in the liver metastasis process of colon cancer. The biological role of these molecules was identified through the GO and KEGG analysis. Moreover, we identified that the molecules SERPINA3, SERPINA1, MMP3, ALDH1A3, PBK and CXCL14 were the independent factors for patients survival. The CXCL14 was selected for further analysis for its most significant P value. Single-cell analysis showed that the CXCL14 was mainly expressed in the fibroblasts. Meanwhile, the biological role of fibroblasts in the colon cancer microenvironment was investigated. Further, the clinical role of CXCL14 in colon cancer was also explored. The result showed that the CXCL14 is a protective factor against colon cancer independent of other clinical parameters like age, gender, clinical stage, and TNM classifications. Then, biological enrichment analysis indicated that the CXCL14 is predominantly involved in the activating of the WNT/β/catenin pathway, pancreas beta cells, peroxisome and bile acid metabolism. Immune infiltration analysis showed that for the patients with high CXCL14 levels, the plasma B cells, CD8 + T cells, neutrophil and NK cells might infiltrate more, in contrast to B cells, monocyte and macrophages. Furthermore, we found that the patients with low CXCL14 expression might be more sensitive to etoposide, rapamycin and sunitinib.

Our study identified CXCL14 as a protective factor in colon cancer, which is mainly expressed in fibroblasts. Multiple studies have explored the role of CXCL14 in cancers. For instance, Xu et al. noticed that CXCL14 is primarily expressed in a novel cell subpopulation and exerts as a key factor in lymph node metastasis in breast cancer [33]. Parikh et al. found that in oral cavity squamous cell carcinoma, CXCL14 was specifically expressed in malignant cells in lymph nodes and enhanced the local infiltration of tumor lymphocytes [34]. Zeng et al. revealed that the abnormal Reactive oxygen species could mediate the cell cycle and motility of colon cancer cells through the CXCL14 signaling pathway [35]. Li et al. demonstrated that the upregulation of CXCL14 could enhance the proliferation and lead to a worse prognosis of ovarian cancer [36]. Westrich et al. indicated that the CXCL14 could upregulate the expression MHC-I, therefore regulating antigen-specific CD8 + T-cell responses and suppressing the human papillomavirus-associated head and neck cancer [37]. In our study, we comprehensively explored the role of CXCL14 in colon cancer, indicating that the CXCL14, as a key factor involved in the liver metastasis process, was mainly expressed in fibroblasts and affected cancer progression through multiple manners.

GSEA analysis showed that CXCL14 is predominantly involved in the activating of the WNT/β-catenin pathway, pancreas beta cells, peroxisome, bile acid metabolism, chromatin assembly or disassembly, DNA conformation change, NDA packaging, protein DNA complex, protein DNA complex subunit organization and protein heterodimerization activity. Yan et al. found that liquidambar acid could inhibit the development of colon cancer by suppressing the Wnt/β-catenin signaling [38]. Yue et al. revealed that the interaction between CYTOR and β-catenin could contribute to colon cancer progression and form a corresponding positive feed-forward circuit [39]. Luo et al. noticed that the lncRNA linc01606 could affect ferroptosis cell death and tumor stemness in colon cancer through SCD1-Wnt/β-catenin-TFE3 axis [40]. Interestingly, Ji et al. found that bile acid metabolism might be correlated with liver tumorigenesis [41]. These studies indicated that CXCL14 might affect colon cancer progression and liver metastasis process through the above-enriched pathways.

Our result found that the CXCL14 was mainly expressed in fibroblasts in the colon cancer microenvironment. Peng et al. revealed that the cancer-associated fibroblasts (CAFs) could induce fatty acid catabolism and promote the peritoneal metastasis process of colon cancer [42]. Hu et al. noticed that the exosomes secreted by CAFs could promote colon cancer metastasis and chemotherapy resistance by mediating the EMT process and tumor stemness [43]. Wang et al. demonstrated that the crosstalk between CAFs and exosomal miRNA could facilitate colon cancer metastasis in a CXCL12/CXCR7-dependent manner [44]. Our results showed that CXCL14 was mainly expressed in fibroblasts, making it an underlying potential target for colon cancer.

Although our research is based on high-quality data and analysis, some limitations still need to be paid attention to. Firstly, the population included in this study was predominantly White race. Therefore, the race bias caused by this could reduce the credibility of our conclusion. Secondly, although our research has enriched the regulatory network of CXCL14 in colon cancer, further biological research is still needed.

### Electronic supplementary material

Below is the link to the electronic supplementary material.

Supplementary Material 1

Supplementary Material 2

Supplementary Material 3

## Data Availability

The open-accessed data can be directly downloaded from https://portal.gdc.cancer.gov/; https://www.ncbi.nlm.nih.gov/geo/query/acc.cgi?acc=GSE41258; http://tisch.comp-genomics.org/home/. Based on the reasonable request, all the other data can be obtained from the corresponding author.
