# Supplementary figures and images for: Comprehensive analysis of CXCL14 uncovers its role during liver metastasis in colon cancer
Source: BMC Gastroenterol. 2023 Aug 10;23:273. doi: 10.1186/s12876-023-02896-z (PMC10416425; doi:10.1186/s12876-023-02896-z)

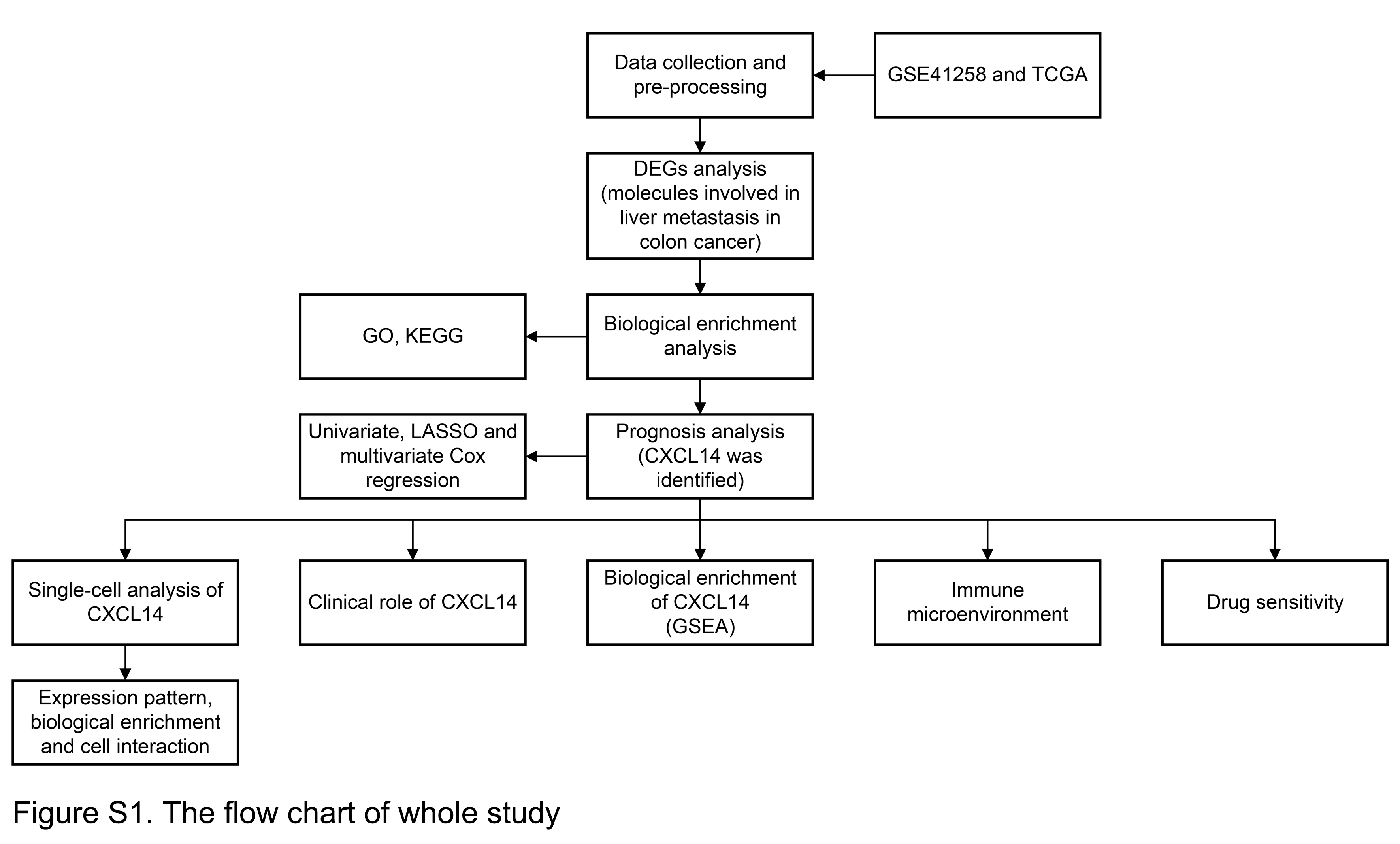

Supplement: Supplementary file 1 — Supplementary Material 1 [file 12876_2023_2896_MOESM1_ESM.tif]

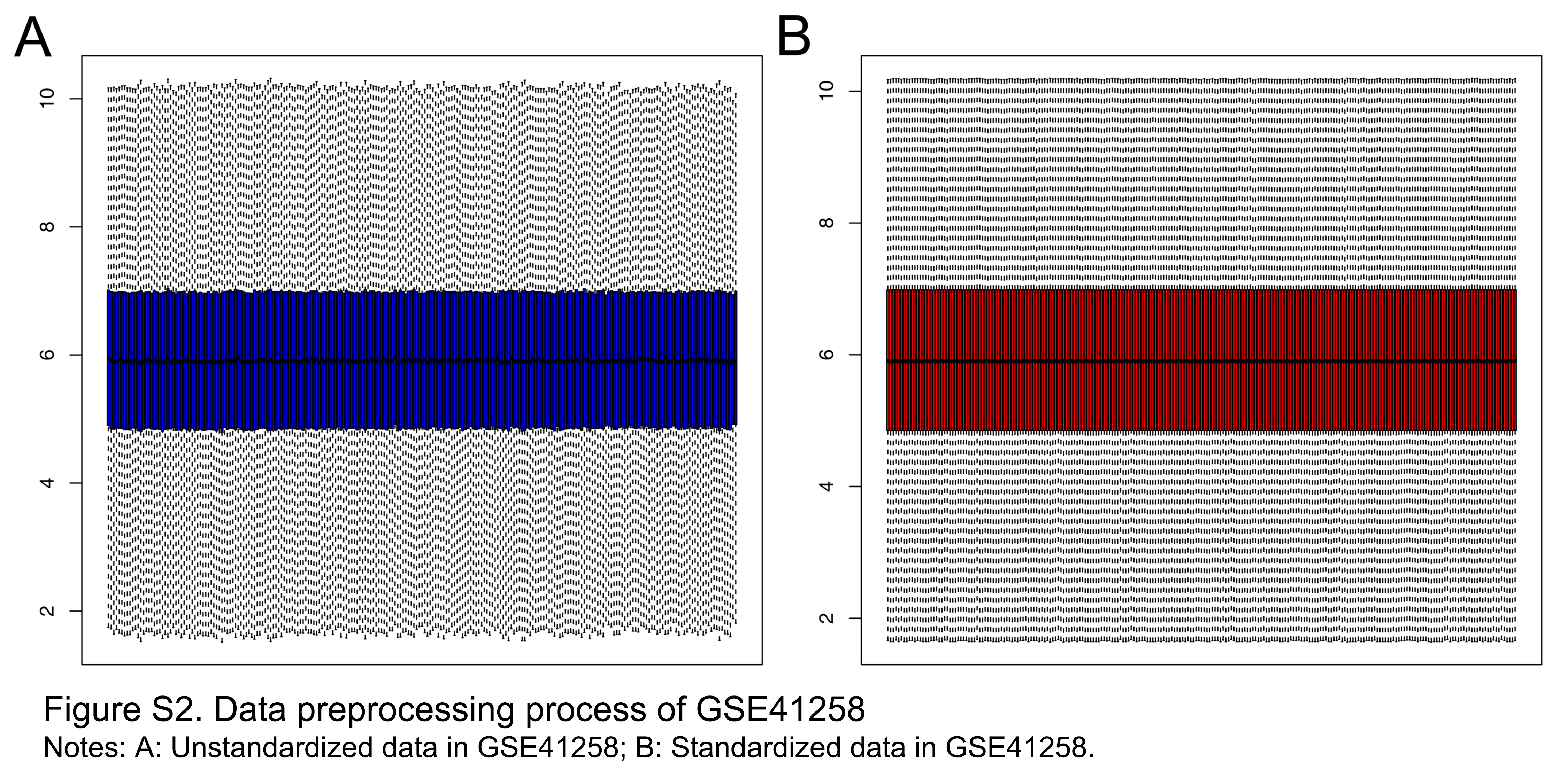

Supplement: Supplementary file 2 — Supplementary Material 2 [file 12876_2023_2896_MOESM2_ESM.tif]

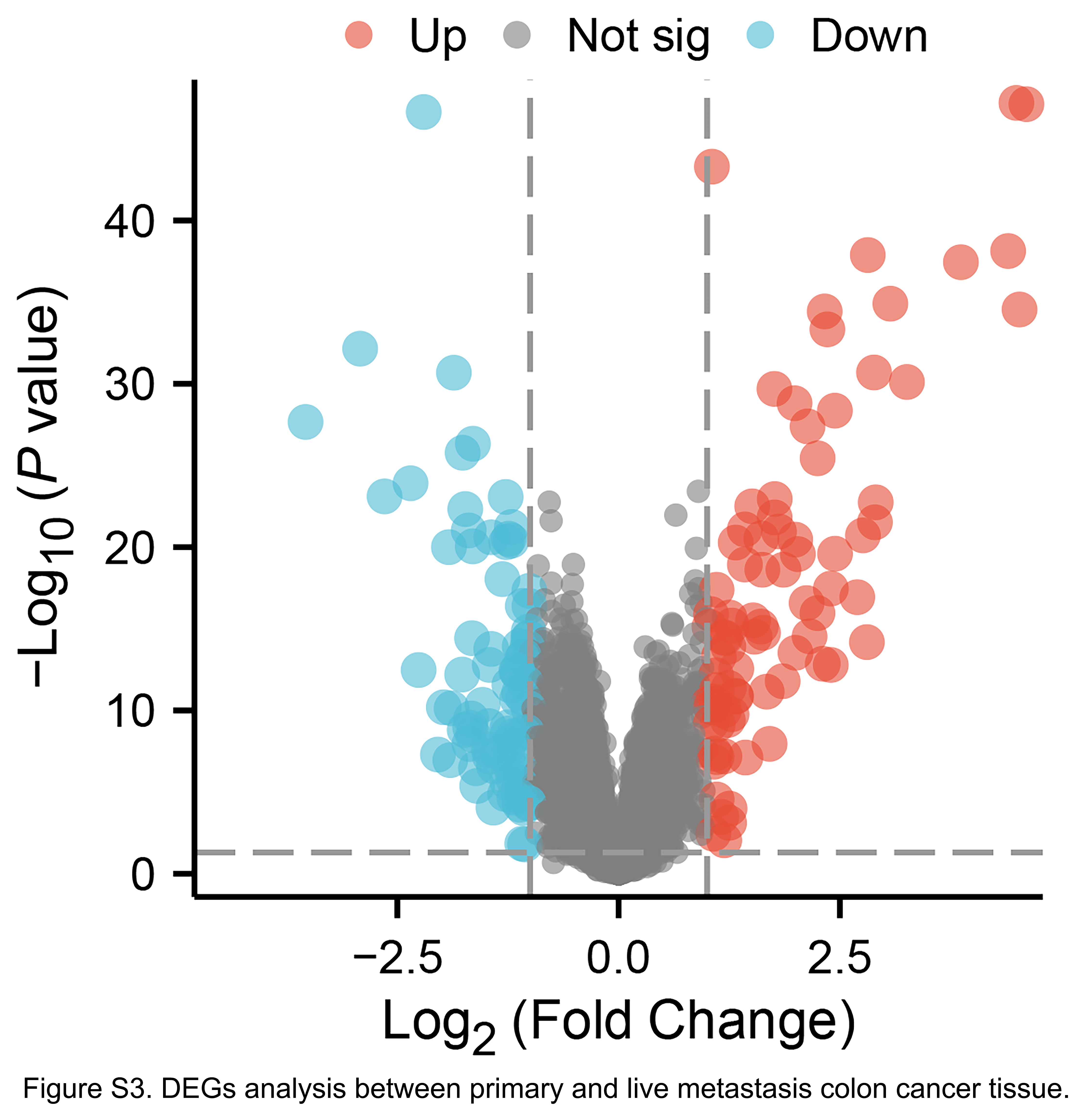

Supplement: Supplementary file 3 — Supplementary Material 3 [file 12876_2023_2896_MOESM3_ESM.tif]
